# Massive left atrial myxoma in pregnancy: case report

**DOI:** 10.1093/ehjcr/ytag229

**Published:** 2026-03-23

**Authors:** Jessica V Yao, Vignesh Ratnaraj, Shivanand Gangahanumaiah, Stacey Peters, Kate English

**Affiliations:** Department of Cardiology, The Royal Melbourne Hospital, 300 Grattan Street, Parkville, Victoria 3052, Australia; Department of Medicine, Dentistry and Health Sciences, The University of Melbourne, Grattan Street, Parkville, Victoria 3010, Australia; Department of Cardiothoracic Surgery, The Royal Melbourne Hospital, 300 Grattan Street, Parkville, Victoria 3052, Australia; Department of Cardiothoracic Surgery, The Royal Melbourne Hospital, 300 Grattan Street, Parkville, Victoria 3052, Australia; Department of Cardiology, The Royal Melbourne Hospital, 300 Grattan Street, Parkville, Victoria 3052, Australia; Department of Medicine, Dentistry and Health Sciences, The University of Melbourne, Grattan Street, Parkville, Victoria 3010, Australia; Department of Cardiology, The Royal Melbourne Hospital, 300 Grattan Street, Parkville, Victoria 3052, Australia; Department of Medicine, Dentistry and Health Sciences, The University of Melbourne, Grattan Street, Parkville, Victoria 3010, Australia

**Keywords:** Cardio-obstetrics, Cardiac tumours, Echocardiography

## Abstract

**Background:**

Cardiac myxomas are rarely diagnosed in pregnancy.

**Case summary:**

A 41-year-old 18+3/40 weeks’ gestation pregnant woman presented with dyspnoea and palpitations on a background of a benign pituitary microadenoma and previous tenosynovial giant cell tumour. Transthoracic echocardiogram demonstrated a 9.1 × 5.5 cm left atrial mass attached to the fossa ovalis with moderate-to-severe obstruction to flow through the mitral valve. After a multidisciplinary discussion regarding maternal and fetal risks, a decision was made to proceed with intrapartum cardiac surgery, given the evidence of mitral inflow obstruction. The mass was successfully excised, and histology confirmed a cardiac myxoma. Postoperative course was uncomplicated, and a healthy baby girl was delivered at 39+1/40 weeks’ gestation.

**Discussion:**

Diagnosis and management of myxomas can be challenging in pregnancy. Symptoms can be misinterpreted as being normal in the setting of physiological changes associated with pregnancy. Surgical excision is necessary to prevent complications, including systemic embolization and haemodynamic deterioration. However, surgery is associated with significant maternal and fetal risks. Thus, a multidisciplinary approach to management is essential.

Learning points

- Transthoracic echocardiogram is an important diagnostic tool in pregnant patients who present with dyspnoea.
- Dyspnoea in pregnancy may be normal due to hormonal and physiological changes. Differential diagnoses include iron deficiency anaemia, pulmonary embolism, and asthma.
- During cardiac surgery in pregnancy, cardiopulmonary bypass should be optimized with blood priming solution, normothermia, high flow rate, pulsatile flow, and high perfusion pressure.
- A multidisciplinary approach to care in women with cardiac disease is paramount.

## Introduction

Left atrial (LA) myxomas are the most common benign cardiac tumours, with an estimated incidence of approximately 0.5 cases per million people.^1^ Myxomas are more common in females and most frequently manifest between the third and sixth decades of life.^2^ Patients most commonly present with dyspnoea, palpitations, and syncope.^3^ Echocardiography is highly specific and sensitive for the diagnosis of myxomas. Cardiac computed tomography (CT) and magnetic resonance imaging (MRI) can also help differentiate myxomas from other cardiac tumours when echocardiographic findings are inconclusive.^3^ Myxomas are extremely rare in pregnancy, with an incidence of approximately 1 in 100 000 pregnancies.^3^ Diagnosis can be challenging, as symptoms, including dyspnoea and palpitations, are often attributed to physiological changes of pregnancy. Transthoracic echocardiography (TTE) is most commonly used to diagnose myxomas in pregnancy.^4^ Transoesophageal echocardiography can also be used, whereas CT and MRI should generally be avoided during pregnancy. Management of cardiovascular disorders in pregnancy requires multidisciplinary input and careful consideration of risks to both the mother and the fetus.^5^

## Summary figure

**Figure:**
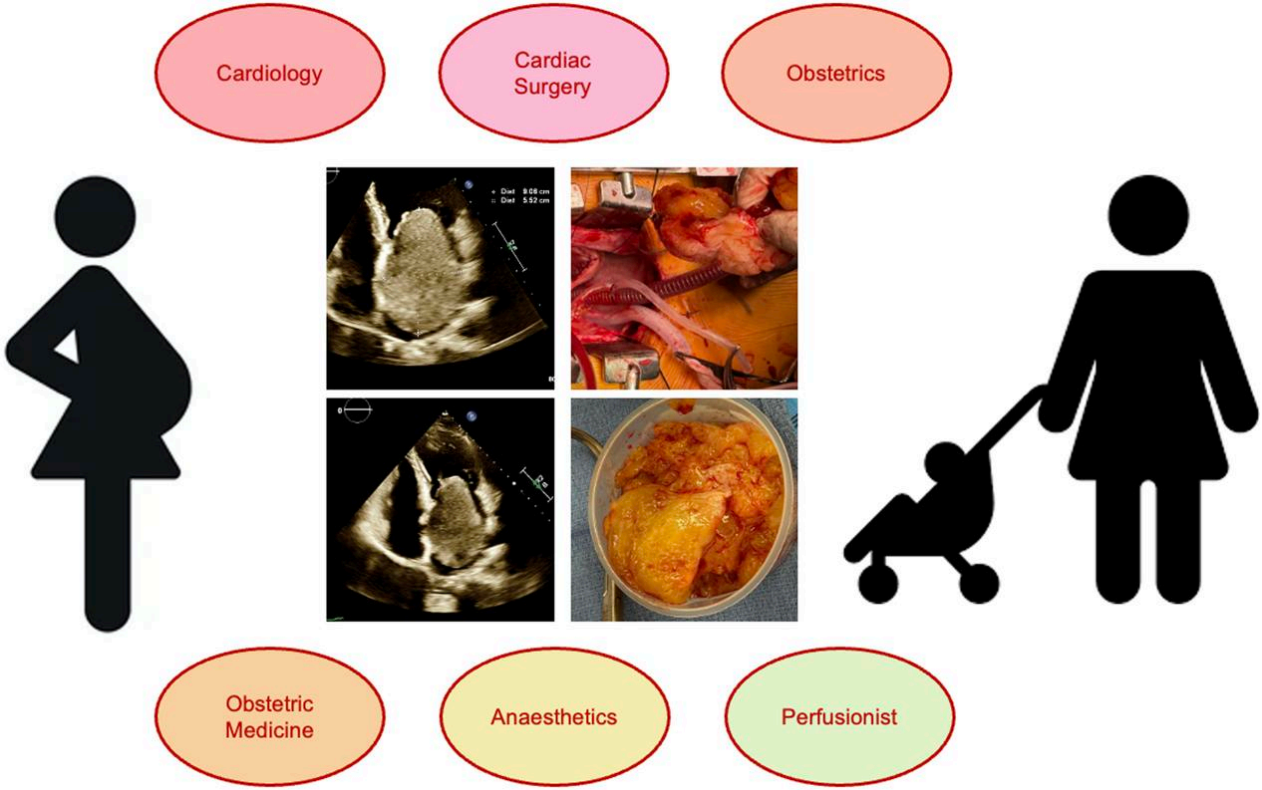


## Case presentation

**Figure 1:**
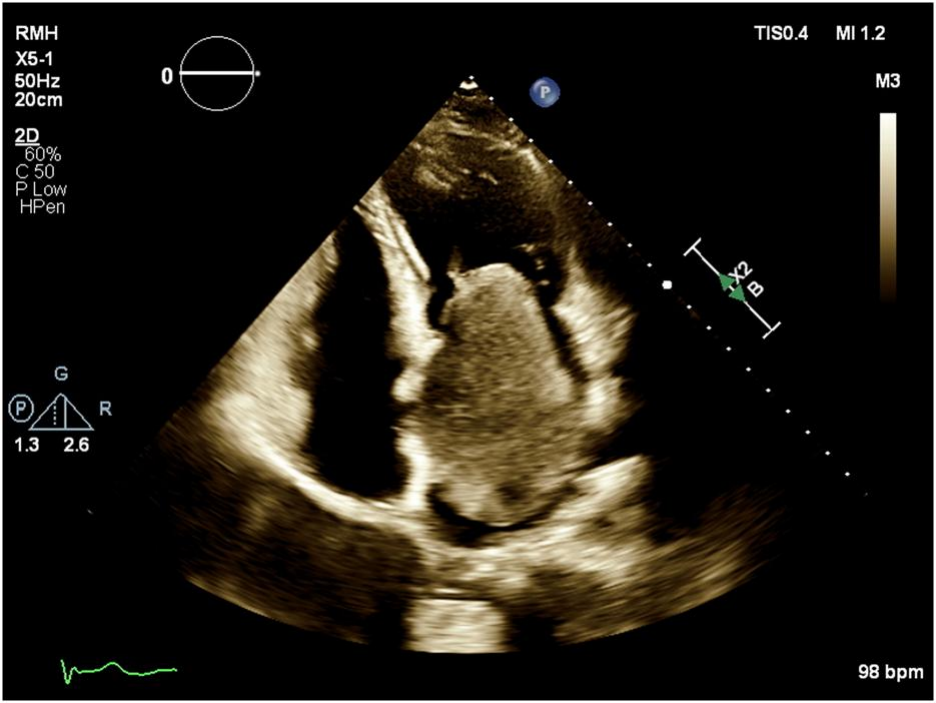
Transthoracic echocardiogram demonstrating an apical 4 chamber view with a massive left atrial mass attached to the fossa ovalis and extending through the mitral valve, causing moderate-to-severe mitral valve obstruction.

**Figure 2:**
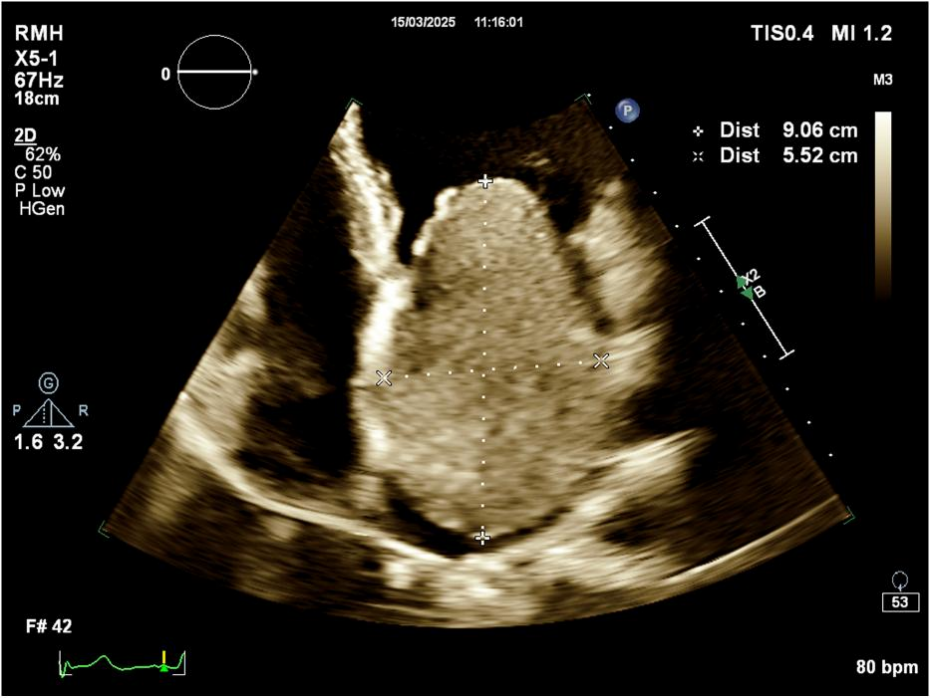
Transthoracic echocardiogram demonstrating the massive left atrial mass which measures 9.1 × 5.5 cm.

**Figure 3:**
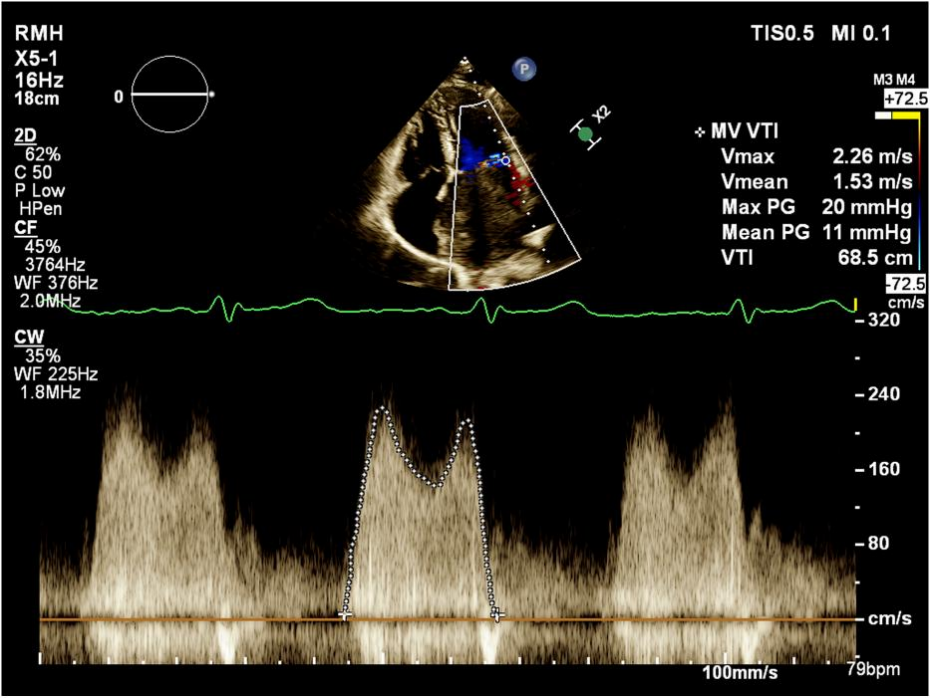
Transthoracic echocardiogram demonstrating massive left atrial mass with moderate-to-severe obstruction to flow through the mitral valve. Continuous wave Doppler across the mitral valve demonstrated a mean gradient of 11 mmHg and a peak gradient of 20 mmHg.

A 41-year-old 18+3/40 weeks’ gestation pregnant designer was reviewed at a routine antenatal clinic appointment. She described exertional dyspnoea, which had progressively worsened over the last 4 years. She also described intermittent palpitations and light-headedness, but no syncope. Her medical history included a benign pituitary microadenoma, for which she was taking cabergoline prior to pregnancy, and a tenosynovial giant cell tumour of her finger that had been surgically excised, as well as migraines, polycystic ovary syndrome, adenomyosis, psoriasis, and eczema. Family history included idiopathic dilated cardiomyopathy in her father. Medications included aspirin 150 mg daily for preeclampsia prophylaxis, folic acid 0.5 mg daily, and a pregnancy multivitamin. She was a lifelong nonsmoker and did not drink alcohol. Obstetric history included two miscarriages and a successful assisted vaginal delivery 5 years prior. On examination, she was normotensive. Heart sounds were dual with a soft diastolic murmur. There were no features of fluid overload. The electrocardiogram demonstrated a sinus rhythm and a normal axis. TTE revealed normal left ventricular function, but a 9.1 × 5.5 cm LA mass (*Figures 1* and *2*, Supplementary material online, *Video S1*) attached to the fossa ovalis, consistent with a LA myxoma. There was moderate-to-severe obstruction to flow through the mitral valve. Mean gradient across the mitral valve was 11 mmHg, and peak gradient was 20 mmHg (*Figure 3*). She was admitted to the hospital and commenced on a heparin infusion, given the high risk of thrombus formation and stroke associated with myxomas.

**Figure 4:**
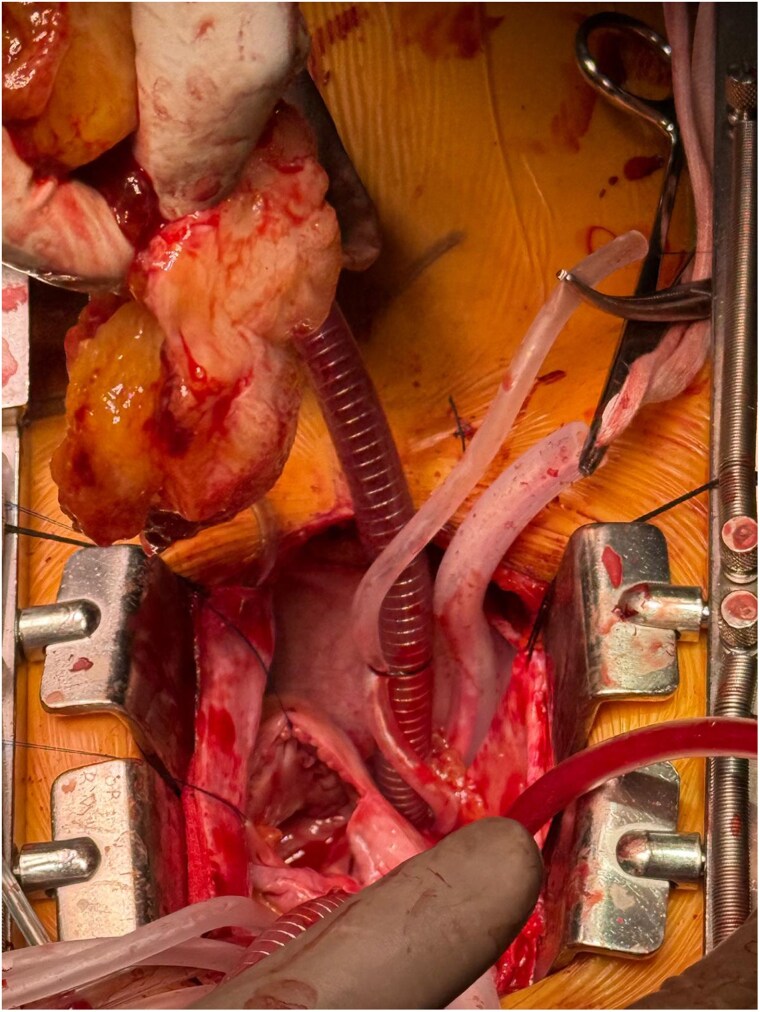
Macroscopic image of left atrial mass, which was successfully excised from the Søndergaard’s groove.

Fetal ultrasound demonstrated a healthy fetus. A multidisciplinary meeting was held between the Cardiology, Cardiothoracic Surgery, Perfusion, Obstetrics, Anaesthetics, and Intensive Care teams. The estimated risk of miscarriage was 25%–30%, with a maternal mortality risk of 3%–4%.^5,6^ After discussion with the patient, the decision was made to proceed with semiurgent surgery, given evidence of mitral inflow obstruction. Preoperative coronary assessment was not performed, as the patient was considered to be at negligible risk for significant coronary artery disease. It was determined that the potential harm related to radiation exposure exceeded the expected benefits. She proceeded to surgery with support from the Cardiac Anaesthetics team. The obstetrics team was also on standby. A median sternotomy was performed. After systemic heparinization, aortobicaval cannulation was performed. Cardiopulmonary bypass was initiated, and normothermia was maintained with pulsatile flow throughout the surgery. The LA mass was noted to be friable, jelly-like, and attached to the fossa ovalis of the interatrial septum, consistent with a myxoma. The mass was excised through Søndergaard’s groove (*Figure 4* and Supplementary material online, *Figure S1*). After close inspection for fragmentation and debris, the interatrial septum was patched with autologous pericardium, and Søndergaard’s groove was closed.

She required minimal vasopressor support and was extubated on the day after surgery. Doppler ultrasound confirmed the presence of a fetal heartbeat with no decelerations. The postoperative course was complicated by several episodes of symptomatic atrial fibrillation with rapid ventricular rate, managed with Metoprolol. Repeat TTE demonstrated an intact interatrial septum, mildly dilated biatrial size, and normal valvular function (see Supplementary material online, *Video S2*). Histology confirmed a benign cardiac myxoma with no evidence of necrosis or invasive growth (see Supplementary material online, *Figures S2* and *S3*). She was discharged home 8 days after her surgery. She remained stable with a normal TTE at 4 weeks postdischarge. Fetal growth remained stable between the 70th–90th percentile, and the fetal echocardiogram was normal. An elective caesarean section was planned due to breech presentation and a history of pelvic organ prolapse. A healthy baby girl was delivered at 39+1/40 weeks’ gestation via successful caesarean section with a spinal anaesthetic. APGAR was 9 at 5 min. Both mother and baby remain well 3 months postpartum. Genetic testing for Carney complex is awaited, given her history of pituitary microadenoma and giant cell tumour.

## Discussion

Myxomas are the most common benign cardiac tumours; 10% of cardiac myxomas are related to Carney complex, which is a rare autosomal dominant genetic disorder affecting the *PRKAR1A* gene. Carney complex is characterized by multiple endocrine neoplasia syndromes, non-endocrine tumours, and abnormal skin pigmentation. In the majority of cases (75%), myxomas arise in the LA and are attached to the fossa ovalis on the interatrial septum. Clinical presentation varies according to size, mobility, and location of the myxoma. Common symptoms include dyspnoea and palpitations^4^ due to intracardiac obstruction, as in our case. Others present with systemic embolization due to thrombus formation and constitutional symptoms due to interleukin-6 (IL-6) production.^1^ In 20% of cases, patients are asymptomatic. Diagnosis and haemodynamic consequences can be established on echocardiography with high sensitivity and specificity. Little is known about the growth rate of myxomas. Surgical excision remains the mainstay of treatment due to the risk of life-threatening emboli, haemodynamic deterioration, and sudden cardiac death.

Myxomas are rarely diagnosed in pregnancy.^4^ Symptoms may manifest in pregnancy due to physiological changes associated with pregnancy, which can worsen the haemodynamic effects of myxomas. Diagnosis and management of myxomas can be challenging in pregnancy. Symptoms such as dyspnoea can often be misinterpreted as normal physiological changes of pregnancy. Echocardiography is an invaluable tool for evaluating cardiac function and identifying structural abnormalities in pregnancy. Echocardiography is also the most commonly used modality for diagnosing cardiac myxomas in pregnancy.^4^ There remains no consensus as to optimal monitoring of cardiac myxomas and timing of cardiac surgery in pregnancy.^7^ Management is typically guided by maternal symptoms and gestational age. Left-sided myxomas are associated with a 25%–30% risk of stroke due to fragmentation of tumour cells or thrombus. This risk is compounded by the hypercoagulable state of pregnancy. Anticoagulation is generally recommended to reduce thromboembolic risk prior to surgical excision^8^; however, current recommendations are largely derived from case reports and retrospective case series. Notably, embolic complications have been reported in up to 27% of patients with atrial myxomas despite anticoagulation therapy.^8,9^ Thus, anticoagulation should not be considered a substitute for surgical excision.

In those with high embolic risk or valvular obstruction, surgery should be performed imminently.^4^ In symptomatic patients without immediate risk, surgery should be considered but may be delayed until the second trimester, as the risk of miscarriage with cardiac surgery is highest in the first trimester. In asymptomatic patients, maternal cardiac risks should be weighed against fetal risks associated with preterm delivery and cardiopulmonary bypass. Some have suggested that if the myxoma is found in the first trimester, surgery should be delayed until the early second trimester.^10^ If the myxoma is diagnosed after the first trimester, surgery should proceed without delay. Cardiopulmonary bypass should be optimized with blood priming solution, normothermia, a high flow rate, pulsatile flow, and high perfusion pressure to improve fetal survival. If the diagnosis is made late in the third trimester, delivery via a caesarean section should precede cardiac surgery.

A multidisciplinary approach to care is essential in pregnant women with cardiac disease in order to optimize outcomes and minimize risks to both the mother and fetus. At a minimum, the team should include a cardiologist, obstetrician, anaesthetist, and perfusionist. Furthermore, a frank discussion with the patient regarding maternal and fetal risks is paramount.

Our case is notable for several reasons. Surgery was performed in the second trimester, whereas LA myxoma surgery during pregnancy is most commonly reported in the third trimester.^4^ Additionally, the tumour measured 9.1 × 5.5 cm, representing the largest cardiac myxoma reported in pregnancy to our knowledge.^11^ Although our patient was older than the average maternal age described in previous reports, both antenatal surgery and subsequent delivery were uncomplicated due to meticulous planning and multidisciplinary care. Limitations of this report include pending genetic testing results and the relatively short duration of the follow-up.

## Conclusion

We describe a rare case of a massive atrial myxoma in pregnancy, which was successfully managed with surgery. Our case highlights the importance of considering cardiac causes of dyspnoea in pregnant women and underscores the diagnostic utility of TTE. Our case emphasizes the intricacies of decision-making in maternal-fetal medicine and the importance of a multidisciplinary team in minimizing risks and optimizing outcomes.

## Lead author biography


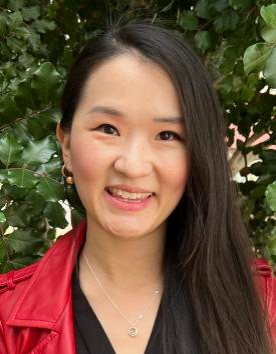


Jessica Yao is a Cardiology Fellow and PhD candidate with a special interest in Cardio-obstetrics and echocardiography.

## Supplementary Material

ytag229_Supplementary_Data

## Data Availability

The data underlying this article are available in this article.

## Notes

**Consent:** The patient has given consent for publication of this case report. This case report complies with the COPE guidelines.
