# Supplementary material for: Massive left atrial myxoma in pregnancy: case report
Source: Eur Heart J Case Rep. 2026 Mar 23;10(4):ytag229. doi: 10.1093/ehjcr/ytag229 (PMC13075940; doi:10.1093/ehjcr/ytag229)
Supplement: ytag229_Supplementary_Data [file ytag229_supplementary_data.zip › Supplementary captions.docx]

**Supplementary figures:**

1. Supplementary Figure 1: Macroscopic image of LA mass consistent with LA myxoma on histology.
2. Supplementary Figure 2: Histology with haematoxylin and eosin stain, demonstrating classic histopathologic features of cardiac myxoma, including neoplastic (lepidic) cells set in a myxoid, paucicellular stroma. The tumour cells are arranged in more complex structures around blood vessels. There is background fresh haemorrhage.
3. Supplementary Figure 3: Histology with haematoxylin and eosin stain, demonstrating classic histopathologic features of cardiac myxoma, including haemorrhage and Gandy-Gamma bodies. The bottom right corner of the image demonstrates tumour attachment to the cardiac muscle.
4. Supplementary Video 1: Initial TTE demonstrating a massive LA mass attached to the fossa ovalis prolapsing through the mitral valve, causing moderate to severe obstruction through the mitral valve
5. Supplementary Video 2: Postoperative TTE demonstrating an intact interatrial septum, mildly dilated biatrial size and normal valvular function
