# Supplementary figures and images for: Massive left atrial myxoma in pregnancy: case report
Source: Eur Heart J Case Rep. 2026 Mar 23;10(4):ytag229. doi: 10.1093/ehjcr/ytag229 (PMC13075940; doi:10.1093/ehjcr/ytag229)

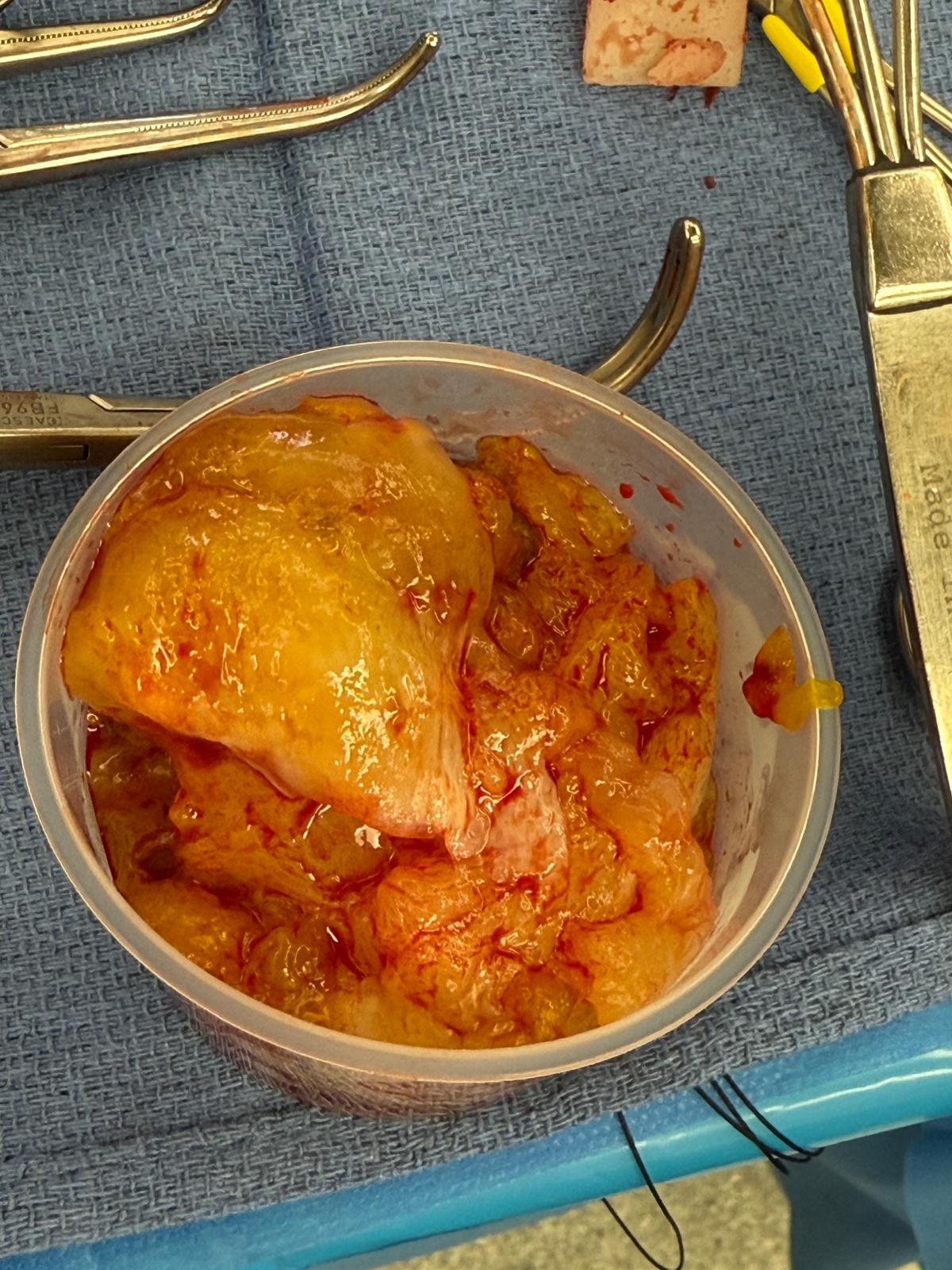

Supplement: ytag229_Supplementary_Data [file ytag229_supplementary_data.zip › Supplementary figure 1.JPG]

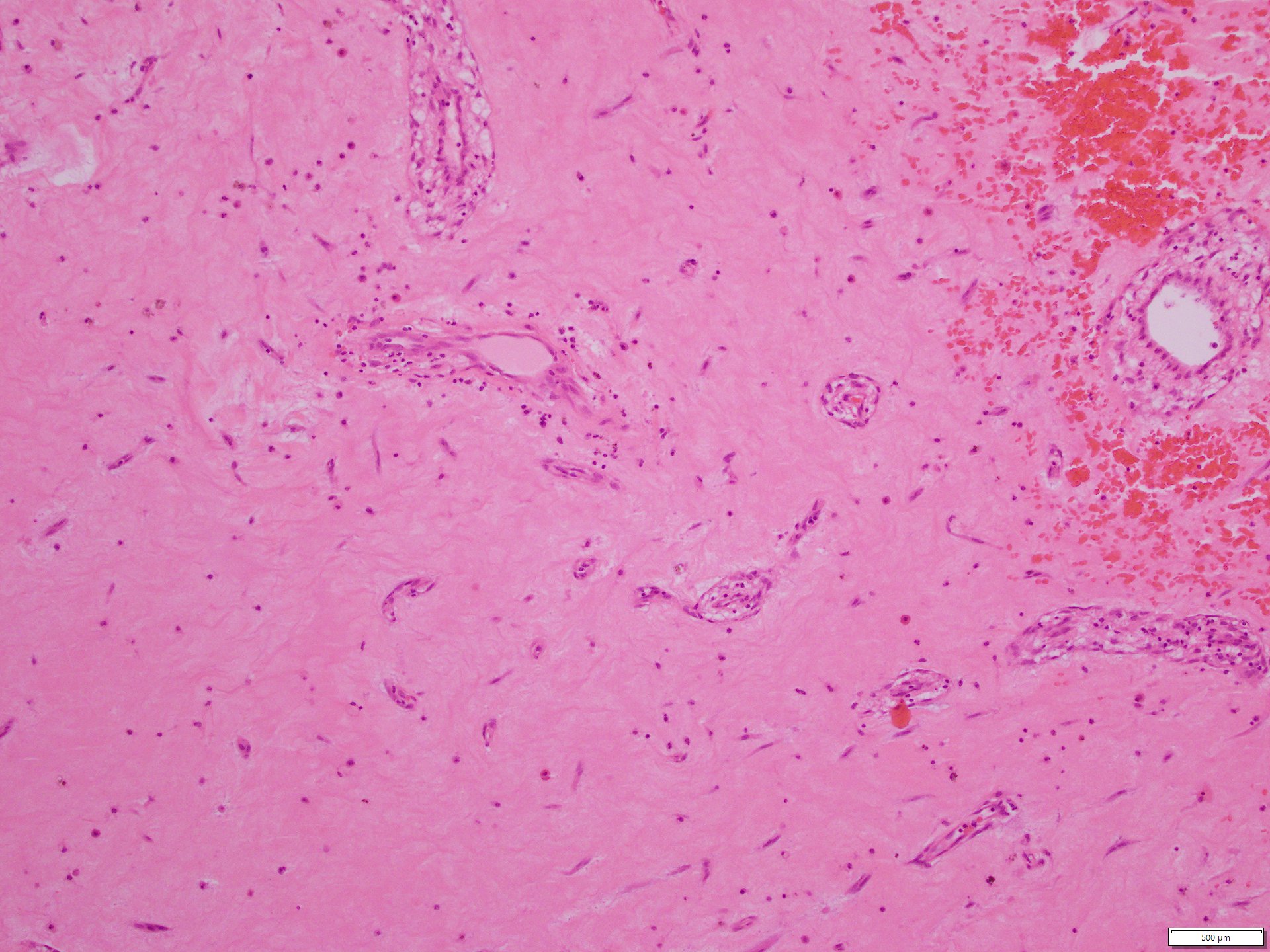

Supplement: ytag229_Supplementary_Data [file ytag229_supplementary_data.zip › Supplementary figure 2.jpg]

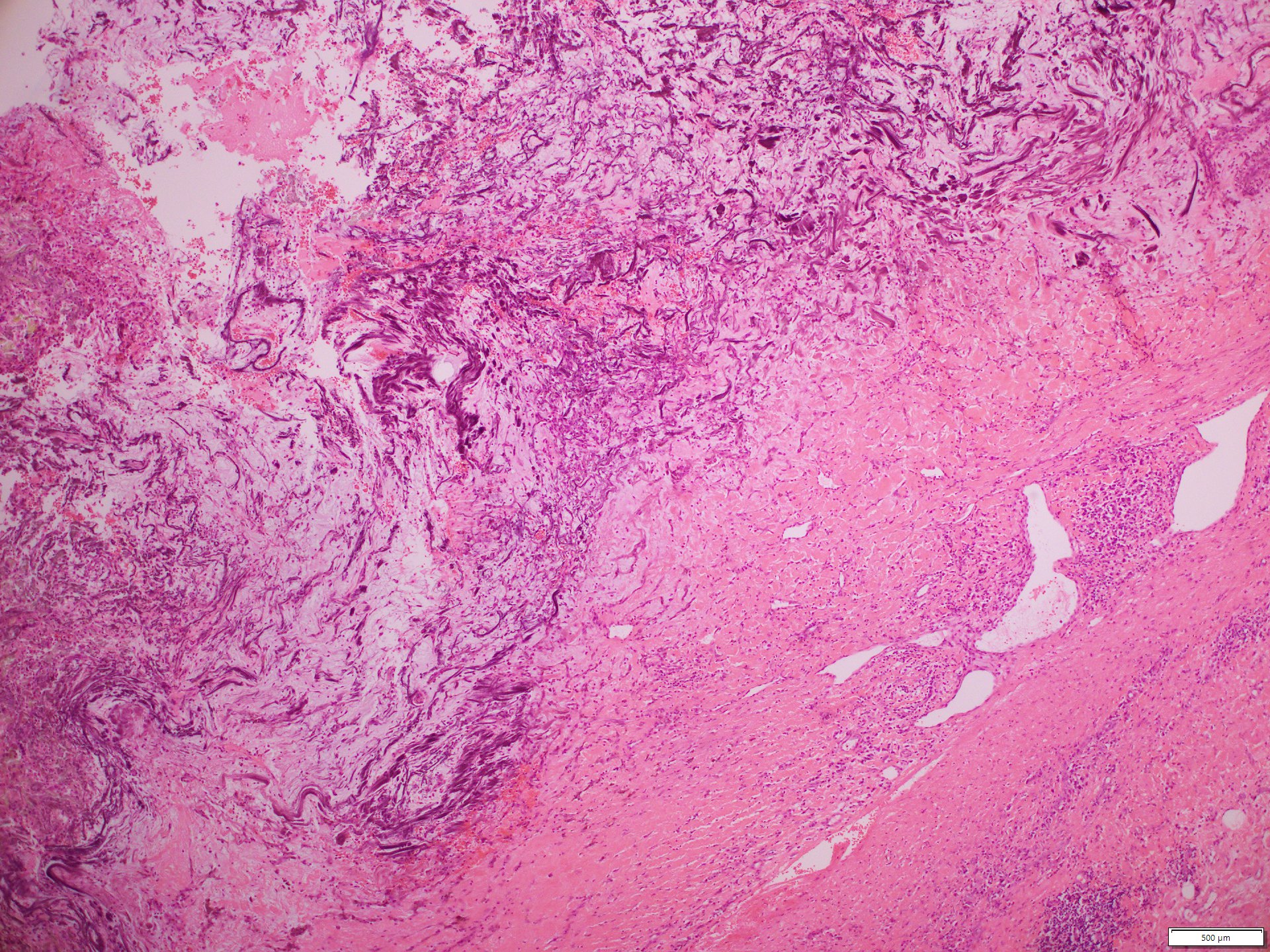

Supplement: ytag229_Supplementary_Data [file ytag229_supplementary_data.zip › Supplementary figure 3.jpg]
